# Supplementary material for: Identification of photoperiod-induced specific miRNAs in the adrenal glands of Sunite sheep (Ovis aries)
Source: Front Vet Sci. 2022 Jul 22;9:888207. doi: 10.3389/fvets.2022.888207 (PMC9354845; doi:10.3389/fvets.2022.888207)
Supplement: Supplementary file 1 [file Data_Sheet_1.ZIP › Supplementary file/miRDeep2 file.zip!/miRDeep2 file/Novel_Structure/LP42A1/Novel_254.pdf]

Provisional ID	:	NC_019477.2_29320
Score total	:	1.1
Score for star read(s)	:	-1.3
Score for read counts	:	0
Score for mfe	:	0.8
Score for randfold	:	1.6
Score for cons. seed	:	
Total read count	:	106
Mature read count	:	106
Loop read count	:	0
Star read count	:	0

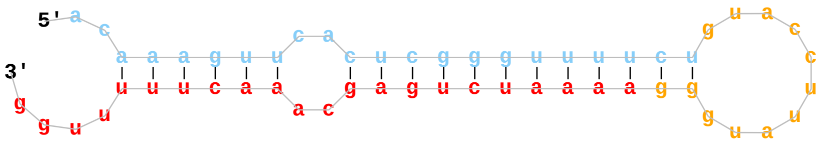

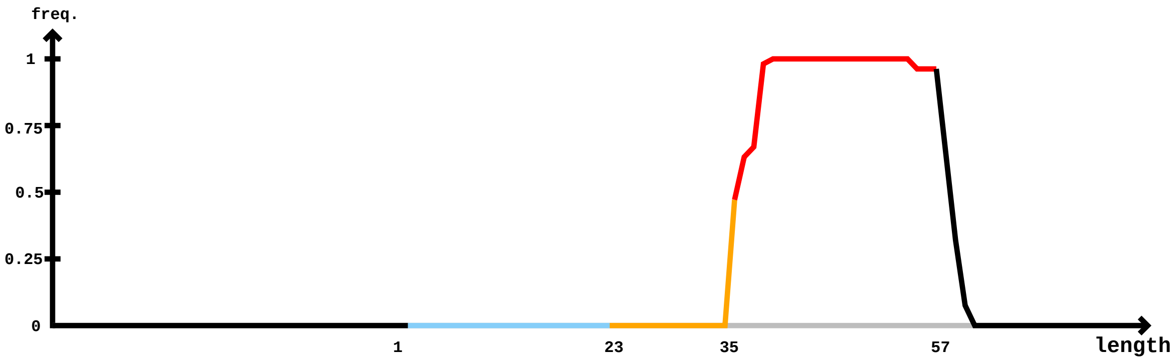

Star Mature

```
5'- uacuugacuuaauuuuuuaauguauucaacuggccacaaaguucacucggguuuucuguaccuuaugggaaaaucugagcaaacuuuuuggccaacccacuauuuucaaaca   -3'    exp
    ...((((....(((....)))....)))).((((((.((((((..((((((((((((.........))))))))))))..)))))).))))))...................    reads      mm          sample
    ....................................................................aaaaucugaAcaaacuuuu.........................    4          1           seq
    ....................................................................aaaaucugaAcaaacuuuuugg......................    45         1           seq
    ....................................................................aaaaucugaAcaaacuuuuuggc.....................    1          1           seq
    .....................................................................aaaucugaAcaaacuuuuugg......................    14         1           seq
    .....................................................................aaaucugagcaaacuuuuuggc.....................    3          0           seq
    ......................................................................aaucugaAcaaacuuuuuggc.....................    4          1           seq
    .......................................................................aucugaAcaaacuuuuuggcc....................    25         1           seq
    .......................................................................aucugaAcaaacuuuuuggcca...................    8          1           seq
    ........................................................................ucugaAcaaacuuuuuggc.....................    1          1           seq
    ........................................................................ucugagcaaacuuuuuggcc....................    1          0           seq
```
